# Proinflammatory mucosal-associated invariant CD8+ T cells react to gut flora yeasts and infiltrate multiple sclerosis brain

**DOI:** 10.3389/fimmu.2022.890298

**Published:** 2022-07-28

**Authors:** Francesca Gargano, Gisella Guerrera, Eleonora Piras, Barbara Serafini, Monica Di Paola, Lisa Rizzetto, Maria Chiara Buscarinu, Viviana Annibali, Claudia Vuotto, Marco De Bardi, Silvia D’Orso, Serena Ruggieri, Claudio Gasperini, Lorenzo Pavarini, Giovanni Ristori, Mario Picozza, Barbara Rosicarelli, Clara Ballerini, Rosella Mechelli, Francesco Vitali, Duccio Cavalieri, Marco Salvetti, Daniela F. Angelini, Giovanna Borsellino, Carlotta De Filippo, Luca Battistini

**Affiliations:** ^1^Neuroimmunology Unit, Istituto di Ricovero e Cura a Carattere Scientifico (IRCCS) Santa Lucia Foundation, Rome, Italy; ^2^Istituto Superiore di Sanità, Department of Neuroscience, Rome, Italy; ^3^Research and Innovation Centre – Fondazione Edmund Mach, S. Michele all’Adige (TN), Italy; ^4^Neurology and Centre for Experimental Neurological therapies (CENTERS), S. Andrea Hospital, Sapienza University, Rome, Italy; ^5^Department of Neuroscience “Lancisi”, S. Camillo Hospital, Rome, Italy; ^6^University of Florence, Department of Biology, Florence, Italy; ^7^University of Florence, Clinical and Experimental Medicine, Florence, Italy; ^8^National Research Council, Institute of Agricultural Biology and Biotechnology, Pisa, Italy

**Keywords:** multiple sclerosis, dysbiosis, mycobiome, MAIT cells, neuroinflammation

## Abstract

The composition of the intestinal microbiota plays a critical role in shaping the immune system. Modern lifestyle, the inappropriate use of antibiotics, and exposure to pollution have significantly affected the composition of commensal microorganisms. The intestinal microbiota has been shown to sustain inappropriate autoimmune responses at distant sites in animal models of disease, and may also have a role in immune-mediated central nervous system (CNS) diseases such as multiple sclerosis (MS). We studied the composition of the gut mycobiota in fecal samples from 27 persons with MS (pwMS) and in 18 healthy donors (HD), including 5 pairs of homozygous twins discordant for MS. We found a tendency towards higher fungal abundance and richness in the MS group, and we observed that MS twins showed a higher rate of food-associated strains, such as *Saccharomyces cerevisiae*. We then found that in pwMS, a distinct population of cells with antibacterial and antifungal activity is expanded during the remitting phase and markedly decreases during clinically and/or radiologically active disease. These cells, named MAIT (mucosal-associated invariant T cells) lymphocytes, were significantly more activated in pwMS compared to HD in response to *S. cerevisiae* and *Candida albicans* strains isolated from fecal samples. This activation was also mediated by fungal-induced IL-23 secretion by innate immune cells. Finally, immunofluorescent stainings of MS post-mortem brain tissues from persons with the secondary progressive form of the disease showed that MAIT cells cross the blood–brain barrier (BBB) and produce pro-inflammatory cytokines in the brain. These results were in agreement with the hypothesis that dysbiosis of the gut microbiota might determine the inappropriate response of a subset of pathogenic mucosal T cells and favor the development of systemic inflammatory and autoimmune diseases.

## Introduction

The composition of the intestinal microbiota plays a critical role in shaping the immune system (1). Modern lifestyle, mindless use of antibiotics, and exposure to pollution have significantly affected the balance of commensal microorganisms in humans. In animal models of disease, the connection between alterations in microbiota composition and altered immunity at distant sites has been shown (2, 3), and in humans, these alterations are thought to be the drivers of the increased proinflammatory and allergic diseases observed in Western countries (4, 5). The mechanistic links between microbiota and autoimmunity include skewing of lymphocyte populations towards proinflammatory subsets, alterations of gut permeability with leakage of microbial products and systemic immune cell activation, and the release of immune-modulating microbial metabolites (6–8).

As several studies have shown, the microbiota is altered in immune-mediated CNS diseases such as MS (9–11). In pwMS, specific bacterial taxa related to proinflammatory responses are favored (12) while short-chain fatty acid (SCFAs)-producing bacteria are reduced (11). Interestingly, researchers are also starting to link MS to a fungal etiology, having revealed the presence of mycotoxins, IL-17, chitotriosidase, and antibodies against fungi (13, 14) in persons with MS (pwMS). Indirectly, alterations of intestinal permeability (IP) have also been detected in relapsing-remitting MS (RR-MS) patients (15), with zonulin being recognized as a peripheral marker of IP and blood–brain barrier (BBB) in MS (16).

These alterations have immunological consequences and may promote the activation and expansion of proinflammatory pathogenic cells involved in MS pathogenesis. The intestinal mucosa is indeed populated by subsets of resident immune cells that participate in the maintenance of gut barrier integrity and of immune homeostasis, and keep the commensal flora under check. MAIT cells constitute a significant proportion of intestinal lymphocytes, and they are mostly CD8+ lymphocytes expressing the semi-invariant TCR Vα7.2 endowed with cytotoxic abilities toward several pathogens (17).

MAIT cells appear to be involved in a wide range of infectious and non-infectious diseases (17) and show specificity for microbial riboflavin-derivative antigens presented by the major histocompatibility complex (MHC) class I–like protein MR1. They exhibit rapid effector responses, similar to innate ones, and can be activated by both T-cell antigen receptor (TCR)-dependent and TCR-independent mechanisms (18).

Seminal studies discovered that derivatives of vitamin B2 and B9 bind to MR1 and that vitamin B2 derivatives are agonists for MAIT cells (19, 20). Since riboflavin synthesis is conserved among bacteria and fungi, MAIT cells respond to a diverse array of microbes. Moreover, studies in animals treated with antibiotics or in germ-free conditions have shown that among all lymphocyte subsets, MAIT cells are the most impacted by the absence of the microbiota (21).

The activation of MAIT cells by several bacteria (22, 23), virus (24, 25), and fungi (17, 26) has been shown in several studies. Here, we set out to characterize the microbiota from fecal samples from HD and pwMS, including a rare cohort of homozygous twins discordant for MS, and we studied the composition of the fungal fraction. The fungal communities showed higher abundance and richness in the MS group. We also find that MAIT cells from pwMS are significantly more activated and have higher proliferative responses to exposure to fungal strains than those from HD, and that MAIT cell activation and proliferation are mediated predominantly by IL-23 produced by innate immune cells.

Finally, we show that MAIT cells can cross the BBB and produce pro-inflammatory cytokines in the brain. These results support the hypothesis that dysbiosis of the gut microbiota may determine a dysfunction of mucosal responses and may favor the development of systemic inflammatory and autoimmune diseases.

## Results

### Cultivable fungal isolates from fecal samples of pwMS and HD

To characterize the cultivable mycobiota associated with MS, we selected, among the enrolled volunteers, 27 pwMS and 21 HD. By using selective media with bacterial growth inhibitors, we obtained a total of 2,000 fungal isolates from fecal samples of 22 out of 27 pwMS (81.4%) and of 15 out of 21 HD (71.4%). The MS group showed a higher number of isolates compared to HD (*N* = 1,608 isolates from MS and *N* = 392 isolates from HD). In these cohorts, 5 pairs of monozygotic twins discordant for MS disease were included (5 MS twins and 5 HD twins), and we obtained 620 fungal isolates from 5 MS twins and 270 isolates from 3 HD twin. By amplification and sequencing of the rDNA ITS1-5.8S-ITS2 [ribosomal Internal Transcribed Spacer (ITS)] region, with universal fungal primers (ITS1 and ITS4), we identified 47 different fungal species belonging to 24 different genera (**Table 1**).

**Table 1:** Quantification of cultivable fungal species from MS and HD stool samples.

Species	No. of isolates				No. of subjects		Abundance value correction	
	MS	HD	% on totalMS isolates	% on totalHD isolates	MS	HD	MS	HD
*Candida albicans*	388	30	24.1%	7.7%	9	7	124.71	14.00
*Candida parapsilosis*	196	19	12.2%	4.8%	4	4	28.00	5.07
*Saccharomyces delbrueckii*	185	2	11.5%	0.5%	2	2	13.21	0.27
*Saccharomyces cerevisiae*	99	1	6.2%	0.3%	3	1	10.61	0.07
*Pichia membranifaciens*	200	0	12.4%	0.0%	1	0	7.14	0.00
*Exophiala dermatitidis*	76	50	4.7%	12.8%	2	1	5.43	3.33
*Candida zeylanoides*	123	0	7.6%	0.0%	1	0	4.39	0.00
*Aspergillus pseudoglaucus*	15	55	0.9%	14.0%	7	1	3.75	3.67
*Candida glabrata*	84	1	5.2%	0.3%	1	1	3.00	0.07
*Exophiala phaeomuriformis*	73	0	4.5%	0.0%	1	0	2.61	0.00
*Pichia manshurica*	70	12	4.4%	3.1%	1	2	2.50	1.60
*Rhodotorula mucilaginosa*	8	15	0.5%	3.8%	6	3	1.71	3.00
*Penicillium commune*	11	0	0.7%	0.0%	4	0	1.57	0.00
*Aspergillus ruber*	19	0	1.2%	0.0%	2	0	1.36	0.00
*Galactomyces candidum*	16	199	1.0%	50.8%	1	1	0.57	13.27
*Trichosporon sp*	12	0	0.7%	0.0%	1	0	0.43	0.00
*Aspergillus terreus*	2	1	0.1%	0.3%	2	1	0.14	0.07
*Corynascus sepedonium*	4	0	0.2%	0.0%	1	0	0.14	0.00
*Pichia fermentans*	3	0	0.2%	0.0%	1	0	0.11	0.00
*Pyrenochaeta unguis hominis*	3	0	0.2%	0.0%	1	0	0.11	0.00
*Pichia kudriavzevii*	2	0	0.1%	0.0%	1	0	0.07	0.00
*Candida orthopsilosis*	1	0	0.1%	0.0%	1	0	0.04	0.00
*Chaetomium brasiliense*	1	0	0.1%	0.0%	1	0	0.04	0.00
*Chaetomium globosum*	1	0	0.1%	0.0%	1	0	0.04	0.00
*Cryptococcus albidus*	1	0	0.1%	0.0%	1	0	0.04	0.00
*Cryptococcus diffluens*	1	0	0.1%	0.0%	1	0	0.04	0.00
*Dipodascaceae family isolate*	1	0	0.1%	0.0%	1	0	0.04	0.00
*Geotrichum candidum*	1	0	0.1%	0.0%	1	0	0.04	0.00
*Malassezia globosa*	1	0	0.1%	0.0%	1	0	0.04	0.00
*Penicillium carneum*	1	0	0.1%	0.0%	1	0	0.04	0.00
*Penicillium chrysogenum*	1	0	0.1%	0.0%	1	0	0.04	0.00
*Penicillium echinulatum*	1	0	0.1%	0.0%	1	0	0.04	0.00
*Penicillium expansum*	1	0	0.1%	0.0%	1	0	0.04	0.00
*Penicillium infrapurpureum*	1	0	0.1%	0.0%	1	0	0.04	0.00
*Phoma sp*	1	0	0.1%	0.0%	1	0	0.04	0.00
*Talaromyces stollii*	1	0	0.1%	0.0%	1	0	0.04	0.00
*Thelebolus ellipsoideus*	1	0	0.1%	0.0%	1	0	0.04	0.00
*Thielavia sp*	1	0	0.1%	0.0%	1	0	0.04	0.00
*Trichosporon ovoides*	1	0	0.1%	0.0%	1	0	0.04	0.00
*Wickerhamomyces anomalus*	1	0	0.1%	0.0%	1	0	0.04	0.00
*Candida lusitaniae*	0	1	0.0%	0.3%	0	1	0.00	0.07
*Debaryomyces hansenii*	0	1	0.0%	0.3%	0	1	0.00	0.07
*Pichia kluyveri*	0	1	0.0%	0.3%	0	1	0.00	0.07
*Rhizopus oryzae*	0	1	0.0%	0.3%	0	1	0.00	0.07
*Rhodosporidium kratochvilovae*	0	2	0.0%	0.5%	0	2	0.00	0.27
*Trichosporon guehoae*	0	1	0.0%	0.3%	0	2	0.00	0.13
**Total no. of isolates**	**1,608**	**392**						

The use of bold letters is instrumental to improve the presentation of the main outcomes.

Considering the absolute abundances, the most abundant fungal species in the MS group were *Candida albicans* (*N* = 388 isolates; 29% of the total number of isolates in MS group)*, Pichia membranifaciens* (*N* = 200; 12.4%), *Candida parapsilosis* (*N* = 196 isolates; 12.2%), *Saccharomyces delbrueckii*, recently classified as *Torulaspora delbrueckii* (*N* = 185; 23%)*, Candida zeylanoides* (*N* = 123; 7.6%), *Saccharomyces cerevisiae* (*N* = 99; 6.15%), and *Candida glabrata* (*N* = 84; 5.2%; **Table 1**). In HD, we found abundance of *Galactomyces candidum* (*N* = 199; 50.7%)*, Aspergillus pseudoglaucus* (*N* = 55; 14%), *Exophiala dermatiditis* (*N* = 50; 12.7%), *C. albicans* (*N* = 30; 7.7%), and *C. parapsilosis* (*N* = 30 isolates; 4.4%; **Table 1**).

These fungal species were previously found as commensal or opportunistic pathogens in the gastrointestinal (GI) tract and in other human body sites (27–29). Regarding the frequency of fungal isolation in MS and HD groups, *C. albicans* was the fungal genus more frequently isolated in both MS and HD groups (**Table 1**; *N* = 9 pwMS and *N* = 7 HD subjects, respectively). *A. pseudoglaucus* and *Rhodotorula mucilaginosa* were the fungal genera more frequently isolated in the MS group, while it was *C. parapsilosis* in both MS and HD groups, although more abundant in MS (**Table 1**).

Considering the different abundances of fungal genera and species, statistical analysis by pairwise Wilcoxon rank-sum test did not show significant differences of enrichment of a specific species between groups, also with respect to the twin subgroups. Yet, the overall number of fungal isolates from pwMS was several-fold higher than that from HD. In the twin subgroups, very few isolates (*N* = 5) were obtained from samples of HD twins, while MS twins showed high counts of a limited number of fungal species, such as *S. cerevisiae* (*N* = 96 isolates) in 2 twin patients, or *T. delbrueckii* (*N* = 170 isolates), *C. zeylanoides* (*N* = 123 isolates), and *C. albicans* (*N* = 4), showing evidence for fungal overgrowth and in general clonal colonization of the patient.

The colonization of different patients with high numbers of different yeasts can potentially be misleading, and it is thus important to always consider the number of patients that contribute to the mean abundance of a species in the population. To facilitate the interpretation and the generalization of the results, we corrected the number of isolates discovered in a class (fungal species) for the ratio of the number of subjects in which we isolated fungal species and the total number of subjects in each group (**Table 1**). This correction provided indication on the most abundant species in the pwMS group and confirmed our observation of a higher fungal abundance in the pwMS group (**Table 1**). Collectively, these data indicated that in pwMS, the most frequently observed genera were *Candida* and *Saccharomyces*, so we focused the ensuing immunological studies on these fungi.

### Metagenomics analysis of the gut mycobiota in pwMS

Several disease states in humans have been recently associated to fungal dysbiosis (27, 30). Thus, we characterized the structure of the gut mycobiota by metagenomic analysis on selected pwMS and HD individuals (20 MS and 18 HD) through high-throughput sequencing of the ribosomal Internal Transcribed Spacer (ITS1) region. We identified 340 operational taxonomic units (OTUs) at the species level.

**Figure 1:**
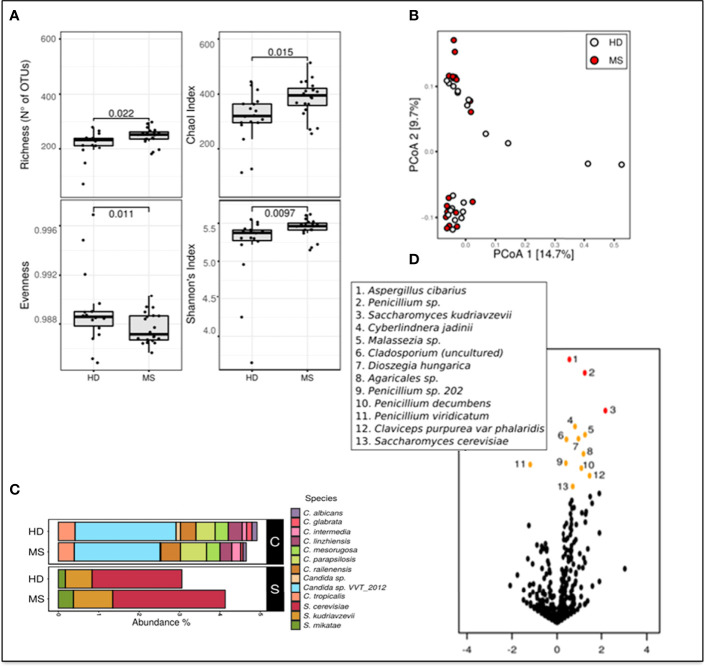
Metagenomic analyses of the gut fungal microbiota in pwMS and HD. **(A)** Alpha diversity measures in pwMS and HD groups. Box plots of observed OTUs (Operational Taxonomic Units, which measure diversity), Chao 1, Evenness, and Shannon indices (*p*-value by Wilcoxon rank sum test). **(B)** Beta diversity analysis. PCoA based on Bray–Curtis distances. **(C)** Barplot of the relative abundances of *Candida* spp. (indicated in black box “C”) and *Saccharomyces* spp. (black box “S”), in MS and HD groups. **(D)** Volcano plots reporting species-level OTUs found at differential abundance in pwMS with respect to HD. *X*-axes show log2 of abundance ratio (fold change, FC) between pwMS and HD. Species-level OTUs with higher abundance in MS samples have positive log2(FC) values, while negative log2(FC) values indicate higher in HD samples. The *y*-axes show negative log10 of *p*-values (negative binomial Wald test). Red dots indicated *p*-value < 0.01, orange dots indicated *p*-value < 0.05, and black dots indicated fungal taxa not statistically significant (*p*-value > 0.05).

To estimate differences in mycobiota diversity between MS and HD groups, we performed analysis of alpha-diversity (**Figure 1A**). We estimated richness (number of observed OTUs), a measure of the total number of species presents in a community, Chao 1 index (a measure of richness based on number of rare species), Evenness (a quantification of how equal the fungal communities are numerically), and Shannon entropy (a measure of diversity in the community). These estimations confirmed that the gut mycobiota of pwMS is significantly more enriched and diverse compared to the gut mycobiota of HD (Wilcoxon rank sum test; **Figure 1A**). Beta diversity analysis, a measure of variability between the microbial communities, performed by PCoA calculated on Bray–Curtis distances (**Figure 1B**), showed the separation of samples in two subgroups. No other variables, such as gender or age, were able to explain this subdivision (PERMANOVA test).

Metataxonomic analysis at the phylum level showed that Ascomycota (62.9% in MS and 62.9% in HD, average of relative abundance) and Basidiomycota (MS 29.2%; HD 29.1%) were the most abundant phyla, while Zygomycota represent about 1% of the fungal communities in the two groups. A total of 5% of relative abundances were unidentified in both MS (5.7%) and HD (6.0%) groups (Unclassified).

Bar plots in **Figure 1C** show differential abundances of *Candida* spp. between MS and HD groups, and, interestingly, an enrichment of *S. cerevisiae, Saccharomyces kudriavzevii*, and *Saccharomyces mikatae* in pwMS.

We next investigated if the abundance of individual microbial taxa differentiated between pwMS and control subjects (**Figure 1D**). At the species level, the volcano plot highlighted more significantly enriched taxa in pwMS (positive values of FC; *n* = 12) than in HD (negative values of FC; *n* = 1), confirming the previous observation of a richer and more diverse mycobiota in HD control. In pwMS, the species with the higher statistical significance (*p* < 0.01 red dots in **Figure 1D**) were *Aspergillus cibarius*, *Penicillium* sp., and *S. kudriavzevii*, while at *p* < 0.05 (yellow plot; **Figure 1D**), several other fungal species were found, including *S. cerevisiae.*

### Phenotyping of intestinal fungal isolates

Phenotypic characterization was performed on a subset of 25 fungal isolates belonging to *C. albicans* (*N* = 9), *S. cerevisiae* (*N* = 6), *S. delbrueckii* (*N* = 4), *C. parapsilosis* (*N* = 1), *Criptococcus albidus* (*N* = 1), *Debaryomyces hansenii* (*N* = 1), *Pichia kluyveri* (*N* = 1), and *T. ovoides* (*N* = 2), with exclusion of filamentous fungi such as *Aspergillus* and *Penicillum*. We evaluated adaptation and resistance of each isolate to conditions mimicking the human GI tract and virulence-related traits. We tested invasiveness, resistance to oxidative stress, growth to supra-optimal temperatures, ability to hyphal or pseudohyphal formation, and sporulation (see *Materials and Methods*). We observed differential phenotypic characteristics not only at the species level, but also at the strain level, as reported in **Table 2**. In general, all fungal isolates were able to grow at supra-optimal temperatures and resist to oxidative stress, except for one *C. albidus* and one *T. delbrueckii* isolate for the former test, and one *C. albicans* and two *S. cerevisiae* isolates, for the latter. A total of three *C. albicans* isolates and only one *S. cerevisiae* isolate showed ability to form hyphae at 37°C either in minimum (YNB) or maximum medium (YPD), while three *S. delbrueckii* isolates were able to form pseudo hyphae at 27°C in YPD medium. Concerning sporulation, three *S. cerevisiae* and one *C. albicans* isolates were able to sporulate. Regarding invasiveness of culture medium, we observed that four *C. albicans* isolates were very invasive and three *S. cerevisiae* showed moderate ability of invasiveness.

**Table 2:** Phenotypic characteristics and antifungals susceptibility of fungal isolates.

ID strain	Identification by ITS1-4 Sanger sequencing	Growth at supra-optimal temperatures		Hyphal and pseudo-hyphal formation				Sporulation	Invasiveness	Oxidative stress (resistance)
				YNB		YPD				
		40°C	42°C	27°C	37°C	27°C	37°C			
#17 YMS24-1	*C. parapsilosis*	++	++	–	–	–	–	–	+++	+++
**#106 YMS102-11**	***C. albicans***	**++**	**++**	**-**	**+**	**-**	**+**	**-**	**+++**	**+++**
#128 YMS12-3	*C. albicans*	++	++	–	–	–	–	–	+++	++
#18 YMS24-2	*C. albicans*	++	++	–	–	–	–	–	–	–
#25 YMS25-7	*C. albicans*	++	++	+	–	+		–	–	+++
#32 YMS25-14	*C. albicans*	++	++	–	+	–	+	–	+++	+
#38 YMS25-20	*C. albicans*	++	++	–	+	–	+	–	+++	+++
**#45 YMS25-27**	***C. albicans***	**++**	**++**	**-**	**-**	**-**	**-**	**-**	**-**	**+**
#51 YMS27-5	*C. albicans*	++	++	–	–	–	–	–	–	+++
#6 YMS19-6	*C. albicans*	++	++	–	–	–	–	++	–	++
#63 YMS28-7	*Cryptococcus albidus*	–	–	–	–	–	–	–	–	+
#120 YSM-8-2	*Debaryomyces hansenii*	++	++	–	–	–	–	–	–	+++
#119 YSM-8-1	*Pichia Kluvery*	++	++	–	–	–	–	–	–	+++
**#2 YMS19-2**	***S. cerevisiae***	**++**	**++**	**-**	**-**	**-**	**-**	**++**	**-**	**++**
#3 YMS19-3	*S. cerevisiae*	++	++	–	+	–	+	++	–	+
**#12 YMS20-2**	***S. cerevisiae***	**+**	**-**	**-**	**-**	**-**	**-**	**-**	**++**	**+**
#13 YMS20-3	*S. cerevisiae*	++	++	–	–	–	–	–	–	–
#14 YMS20-4	*S. cerevisiae*	++	++	–	–	–	–	++	+	+++
#136 YMS18-1	*S. cerevisiae*	++	++	–	–	–	–	++	+	–
#113 YMS7-1	*T. delbrueckii*	+	–	–	–	–	–	–	+	+ +
#114 YMS7-2	*T. delbrueckii*	++	++	–	–	–	–	–	–	++
#117 YMS7-5	*T. delbrueckii*	++	++	+	–	+ (pseudo hyphae)	–	++	–	+++
#118 YMS7-6	*T. delbrueckii*	++	++	–	–	–	–	–	–	+++
#115 YMS7-3	*T. ovoides*	++	++	+	+	+ (pseudo hyphae)	+ (pseudo hyphae)	–	+++	+++
#111 YMS1-1	*Trichosporon* sp.	++	++	–	+	+ (pseudo hyphae)	+	–	+++	++

Qualitative indicators of colony growth, + is used to indicate suboptimal-delayed growth, ++ is used to indicate normal growth, +++ is used to indicate good growth, - is used to indicate absence of growth.

The use of bold letters is instrumental to improve the presentation of the main outcomes.

### Activation of MAIT cells by exposure to yeast extracts

The higher number of diverse fungal isolates obtained from pwMS induces to hypothesize that a common feature of different fungi is associated to disease. To explore this possibility, we selected four isolates belonging to the fungal species *C. albicans* (CA) (*SM106 and SM45* strains) and *S. cerevisiae* (SC) (*SC2 and SC12* strains) (**Table 2**), which showed different phenotypic characteristics and were overrepresented in the pwMS group, so we investigated immune responses to these strains.

**Figure 2:**
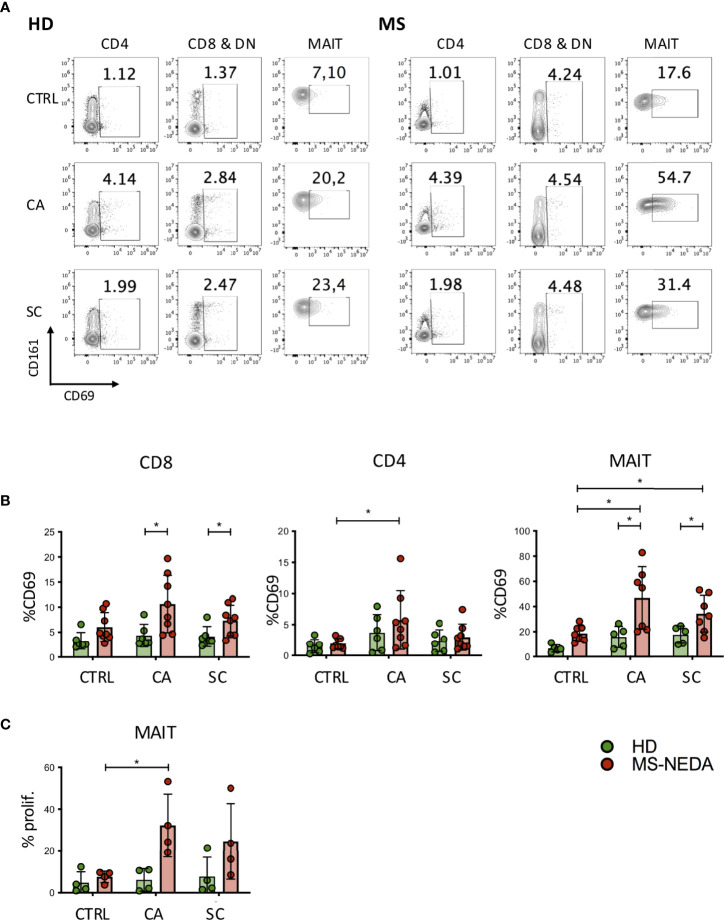
Activation and proliferation of MAIT cells by fungal extracts. PBMCs were incubated for 24 h with SC and CA extracts. At the end of the incubation, the cells were labeled with CD3, CD4, CD8, Vα7.2, CD161, and CD69 and analyzed on a flow cytometer. **(A)** Representative plot of CD69 expression measured on the different cell populations indicated. **(B)** Cumulative date of activation of CD69 (HD *n* = 6; NEDA MS *n* = 8). Bars represent mean with SD. **(C)** MAIT cell proliferation following incubation with CA and SC. PBMCs from pwMS and HD were stained with CFSE and incubated for 5 days in the presence of the fungal extracts. On the fifth day, the cells were collected and stained with CD3, CD8, CD161, and Vα7.2, and the percentage of proliferating cells was measured based on the dilution of CFSE (*n* = 4). Bars represent mean with SD. Statistical significance was assessed by unpaired (between groups) or paired (between experimental conditions) *t*-test. *p < 0.05.

The involvement of T lymphocytes in immunity towards SC and CA was studied through the measurement of CD69 expression, a marker of T-cell activation, on the surface of T cells exposed to these fungi. Freshly isolated peripheral blood mononuclear cells (PBMCs) were cultured in the presence of SC and CA extracts *in vitro*, and CD69 upregulation was measured after 24 h by FACS analysis. As shown in **Figures 2A, B**, both CD4+ and CD8+ T cell subsets show increased levels of CD69 expression following exposure to CA (HD 3.7 ± 3; MS 5.7 ± 4.7 in CD4+ cells, and HD 4.3± 2.2; MS 10.7 ± 5.6 in CD8+ cells) and SC (HD 2.4 ± 1.7; MS 3 ± 2 in CD4+ cells, and HD 4.1 ± 2; MS 7.3 ± 3 in CD8+ and DN cells), with CA being consistently more immune-stimulating than SC both in HD and in pwMS.

We have previously shown that a peculiar subset of CD8+ T cells, characterized by high levels of expression of the surface marker CD161, is expanded in the peripheral blood of pwMS. These cells include most CD8+ IL17-producing T cells, and they also infiltrate the typical lesions in the CNS (31). Interestingly, CD161 high CD8+ T cells correspond to one of the major T-cell populations that patrol the intestinal mucosal, MAIT cells (32, 33). To investigate the possibility that these cells were activated in response to fungal antigens, we measured expression of CD69 also on this subset of cells, following exposure to CA and SC extracts (**Figure 2B**). Interestingly, MAIT cells from pwMS showed significantly increased levels of CD69 expression in response to both CA (HD 15.8 ± 8.5; MS 46.9 ± 24.8) and SC extracts (HD 17.6 ± 6.9; MS 34.4 ± 14.6).

Given the high level of MAIT cell activation following exposure to CA and SC extracts, we also investigated their concomitant proliferative response (**Figure 2C**). Following incubation of freshly isolated PBMCs to the fungal extracts, CD4+ and CD8+ cells did not proliferate (not shown), while MAIT cells from pwMS, but not those from HD, proliferated extensively, particularly in response to CA extracts.

**Figure 3:**
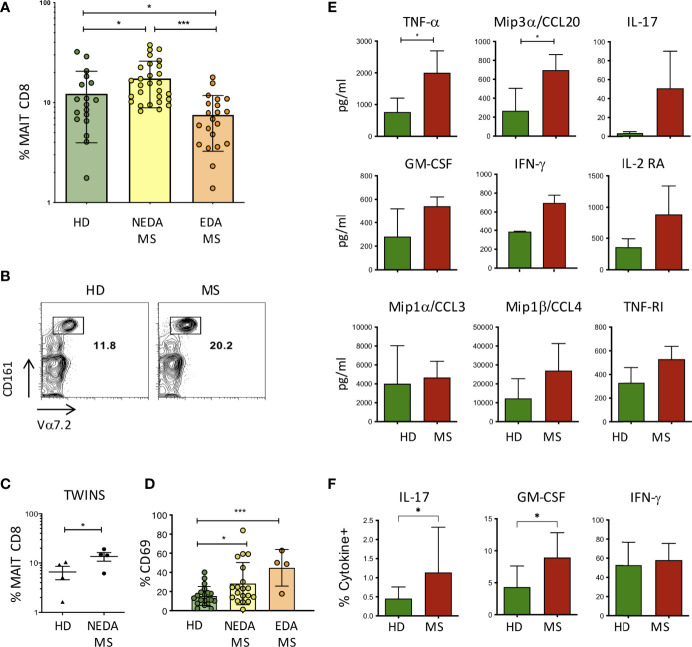
MAIT cells in pwMS and HD. **(A)** Frequency of MAIT cells, measured as a percentage of total CD8+ T lymphocytes, in the peripheral blood of HD (*n* = 18) and in NEDA-MS (*n* = 28) and EDA-MS (*n* = 21) patients. Bars represent mean with SD. **(B)** Representative plot of CD8, Vα7.2, and CD161 positive cells in a pair of twins. **(C)** Cumulative data of MAIT cell frequency in 4 pairs of homozygous twins. Horizontal bars represent mean with SD. **(D)** CD69 expression by MAIT cells measured in freshly isolated PBMC *ex vivo*, in HD (*n* = 20), NEDA-MS (*n* = 19), and EDA-MS (*n* = 4). Bars represent mean with SD. **(E)** Production of cytokines by MAIT cells. CD3+ CD8+ CD161+ Vα7.2 + cells were sorted (purity> 95%) and cultured, then stimulated for 72 h with Dynabeads T-Activator CD3/CD28. The supernatants were then collected and the presence of cytokines in the supernatant was measured with Luminex (*n* = 3). Bars represent SD. **(F)** Cumulative data of IFNγ+ (HD *n* = 22; MS *n* = 28), IL-17A+ (HD *n* = 14; MS *n* = 24), and GM-CSF+ (HD *n* = 22; MS *n* = 28) percentage of MAIT cells following maximal stimulation with PMA and ionomycin of freshly obtained PBMCs from HD and NEDA-pwMS (see **Supplementary Figure 1** for intracellular staining analysis and gate positioning). Bars represent SD. Statistical significance was assessed by unpaired **(A, D–F)** or paired **(C)** t-test. *p < 0.05, ***p < 0.001.

The higher frequency of CA and SC colonies obtained from the feces of pwMS, as well as the vigorous proliferation of MAIT cells in response to the extracts from these fungi, prompted us to investigate whether MAIT cells were more frequent in the peripheral blood of pwMS compared to HD. To this aim, PBMCs were stained with antibodies specific for CD3, CD8, CD161, and TCR Vα7.2, which form the semi-invariant TCR carried by MAIT cells. Indeed, patients with non-evident disease activity (NEDA) presented significantly higher frequencies of MAIT cells compared to HD (**Figure 3A**). Interestingly, in patients with evident disease activity (EDA) as determined clinically or radiologically, the frequency of MAIT cells dropped significantly to levels even lower than those found in healthy subjects. An increased frequency of MAIT cells in the periphery of pwMS was also confirmed in the twin cohort (**Figures 3B, C**).

CD69 expression by MAIT cells was also measured in freshly isolated PBMC *ex vivo*, showing that the activation state of MAIT cells was significantly increased in pwMS. Interestingly, although the frequency of MAIT cells in EDA-MS was lower than that of HD, the level of activation of these cells in relapsing patients is strikingly high (**Figure 3D**).

The effector response of MAIT cells has been shown by us and others (32, 34) to involve the production of proinflammatory cytokines. To confirm these findings also in the setting of the expanded population of MAIT cells in MS, we sorted MAIT cells to purity from HD and from pwMS, and we then stimulated polyclonally through their TCR and we measured cytokine release in the supernatants. After 72 h of stimulation, the screening of multiple cytokines measured with Luminex revealed that MAIT cells produced TNFα, IL-17, IFN-γ, GM-CSF, Mip1α/CCL3, and Mip1β/CCL4, proinflammatory cytokines also involved in the recruitment and activation of monocytes. In addition, we measured the production of IL2-RA and TNF-R1, whose genetic polymorphisms are known to be strongly associated with MS (35, 36), and of Mip3α/CCL20, a chemokine involved in the recruitment of CCR6+ cells across the BBB and into the CNS to recruit CCR6+ cells. MAIT cells from pwMS produced all these soluble factors, although only TNF-α and CCL20 were significantly higher compared to HC (**Figure 3E**).

In order to determine whether a higher fraction of MAIT cells produced proinflammatory cytokines in pwMS compared to HD, we performed intracellular staining for IL-17, GM-CSF, and IFN-γ, coupled with surface staining for CD3, CD8, Vα7.2, and CD161, following maximal stimulation of freshly obtained PBMC with PMA and ionomycin, in the presence of brefeldin. Indeed, we detected an increased percentage of IL-17- and GM-CSF-positive MAIT cells in pwMS, while the fraction of IFN-γ producing cells was equivalent in both groups (**Figure 3F**).

### MAIT cells infiltrate the MS brain

**Figure 4:**
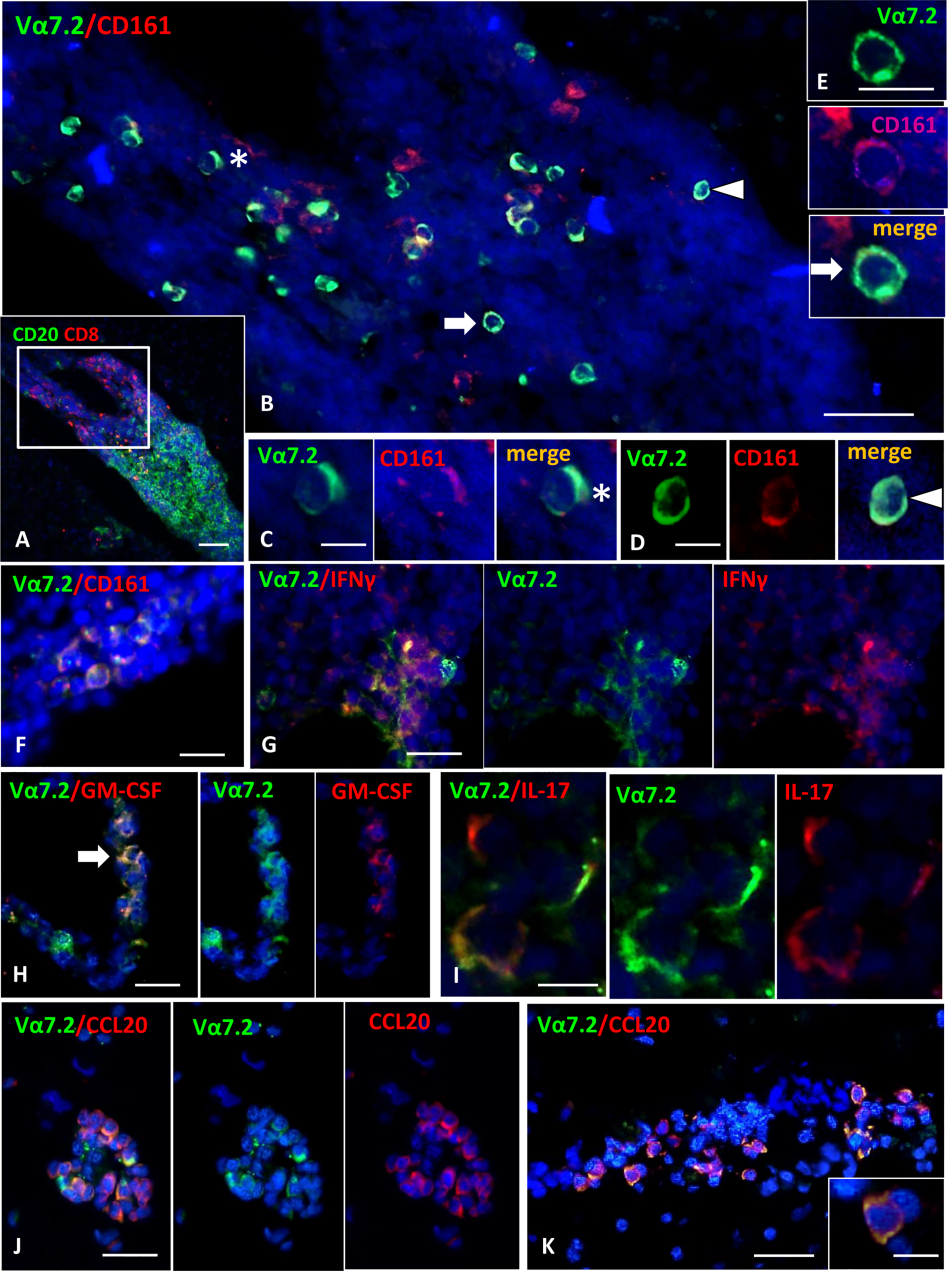
Detection of MAIT cells in the inflammatory infiltrates of MS brain. **(A)** Double staining for CD20 (green) and CD8 (red) shows the presence of a large B cell-enriched perivascular cuff with a compartmentalized CD8+ T cells component in an active WM lesion. **(B)** Double immunofluorescence staining for Vα7.2 (green) and CD161 (red) performed in a serial section reveals numerous Vα7.2+/CD161+ cells within the perivascular infiltrate. These cells are confined to the part of the infiltrate enriched in CD8 (indicated with the square in A). Vα7.2+/CD161+ cells represent about 36% of the CD161+ population present in the infiltrate, and about 90% of the total number of Vα7.2+. **(C–E)** Double-positive cells indicated by asterisk and arrows in panel B are shown at higher magnification. **(F)** Vα7.2+/CD161+ cells are present in a meningeal infiltrate. **(G)** Double immunofluorescence for Vα7.2 (green) and IFNγ (red) shows a group of IFNγ-expressing MAIT cells in an active WM lesion. **(H)** Some Vα7.2+ cells (green) co-expressing GM-CSF (red) are present in a perivascular infiltrate in an active WM lesion. **(I)** Double staining for Vα7.2 (green) and IL-17 (red) highlights the presence of 3 Vα7.2+ MAIT cells expressing IL-17 in an inflammatory infiltrate in the meninges. **(J, K)** Double staining for Vα7.2 (green) and CCL20 (red) in 2 perivascular infiltrates in an active WM lesion. Numerous Vα7.2+ cells co-express CCL20. One double-positive cell is shown in the enlargement in K, where it is possible to distinguish Vα7.2 (green) localized on the cell membrane and CCL20 immunoreactivity (red) in the cytoplasm/membrane. Nuclei are stained with DAPI. Bars: 100 μm in **A**; 50 μm in **B, G, H, J** and **K**; 20 μm in **E** and **F**; 10 μm in **C, D, I**, and inset in **K**.

We next asked whether MAIT cells can be recruited into the inflamed CNS tissue during MS in the progressive phase. To this aim, we performed immunofluorescent stainings for CD8, CD161, and TCR Vα7.2 on post-mortem brain tissues from 15 progressive MS cases, selected for the presence of conspicuous inflammatory infiltrates both in the subarachnoid spaces and in active and chronic active WM lesions. CD8+CD161+Vα7.2+ MAIT cells were detected in the majority (53%) of the MS cases analyzed, and they were almost exclusively localized in areas of massive inflammatory cell infiltration. The percentage of MAIT cells with respect to the total CD8+ T-cell population observed in MS brain tissues analyzed was less than 5%, suggesting that these cells represent a small subset of CD8 T cells infiltrating MS brain. Cells co-expressing CD161/Vα7.2 were observed both in the perivascular spaces of postcapillary venules in active and chronic active WM lesions (**Figures 4A–E**) and in strongly inflamed meninges (**Figure 4F**). Almost all (more than 90%) Vα7.2+ CD161+ cells co-expressed CD8 (data not shown). No CD161+Vα7.2+ cells were observed in inactive lesions, normal-appearing WM, and gray matter. We next performed double immunofluorescence experiments with IL-17, GM-CSF, IFN-γ, and CCL20, to investigate the phenotype of intracerebral MAIT cells. Since virtually all Vα7.2+ cells co-expressed both CD8 and CD161, the immunostaining for TCR Vα7.2 was used in these sets of double labeling experiments to identify MAIT cells. We found a variable number of IFN-γ-expressing Vα7.2+ cells in all cases analyzed, both in inflamed meninges and in some perivascular cuffs in active and chronic active WM lesions (**Figure 4G**); the percentage of total Vα7.2+ cells expressing IFN-γ ranged from 50% (in the meninges) to 80% (in WM lesions). Several intracerebral MAIT cells were found to be positive for IL-17 and GM-CSF, mainly confined to inflamed meninges and active lesions, respectively (**Figures 4H, I**). Interestingly, we found that a significant proportion (ranging from 20% to 60%) of MAIT cells accumulating in the perivascular spaces of highly inflamed blood vessels in active lesions expressed CCL20 (**Figures 4J, K**). Based on these lines of evidence, we can conclude that MAIT cells may cross the BBB and enter in the MS brain, where they could fuel inflammation through the secretion of proinflammatory cytokines. The finding of significant numbers of MAIT cells in active lesions of MS may explain the reduced frequency of these cells in the peripheral blood of EDA pwMS, as has been previously shown in persons with lung infections (17).

### MAIT cell activation by fungi is mediated also by IL-23

**Figure 5:**
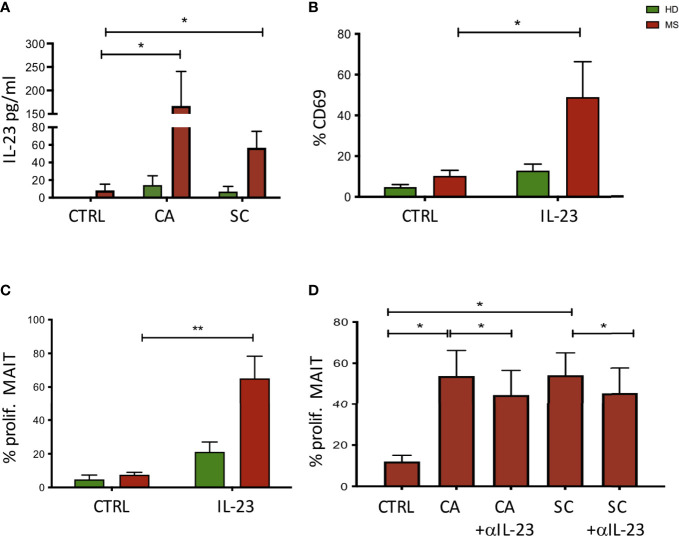
**(A)** Activation of monocyte cells following exposure to fungal extracts. *n* = 9 NEDA pwMS and 9 HD. After stimulation, the supernatants were collected and the cytokine production was measured using ELISA. **(B)** Activation of MAIT cells following exposure to IL-23. Fresh PBMCs were incubated with IL-23, and CD69 expression was measured after 70 h by flow cytometry analysis. *n* = 5 pwMS and 6 HD. **(C)** Proliferation of MAIT cells following exposure to IL-23. Fresh PBMCs were labeled with CFSE for 5 days. After 5 days, cells were collected and MAIT cell proliferation was measured by flow cytometry. *n* = 4 pwMS and 4 HD. **(D)** Proliferation of MAIT cells following exposure to fungal extracts and IL-23 neutralizing antibodies (*n* = 6). Fresh PBMC (*ex vivo*) were incubated with CFSE dilution for 5 days and IL-23 neutralizing antibodies. Cells were then stained and analyzed by flow cytometry. Bars represent mean with SEM. Statistical significance was assessed by paired *t*-test. *p < 0.05, **p < 0.01.

MAIT cells have been shown to be restricted by the antigen-presenting molecule MR1, and to recognize vitamin B metabolites derived from fungi and bacteria (37). Additionally, MAIT cells are activated by viruses indirectly, through the release of IL-12 and/or IL-18 by innate immune cells during a viral infection (38, 39). To investigate the possibility that fungi also activate MAIT cells indirectly, following innate immune cell activation and cytokine production, monocytes were purified from HD and NEDA MS and challenged with CA and SC extracts. Among the tested cytokines (not shown), IL-23 was significantly increased in monocyte cultures stimulated with CA or SC in pwMS compared to HD (**Figure 5A**). Thus, we asked whether IL-23 had a direct effect on the activation of MAIT cells. To this aim, freshly isolated PBMCs were incubated with the cytokine, and CD69 expression was measured after 70 h by FACS analysis. The results show that indeed 50% of MAIT cells from pwMS cells were activated by IL-23, contrary to MAIT cells from HD (**Figure 5B**). Activation by IL-23 resulted not only in CD69 expression, but also in MAIT cell proliferation, as detected by CFSE dilution by flow cytometry. Again, only MAIT cells from pwMS showed significant proliferation (**Figure 5C**), likely due to higher levels of expression of the IL-23R in pwMS compared to healthy donors (**Supplementary Figure 2**).

To confirm the role of IL-23 in the proliferation of MAIT cells induced by fungi, we added IL-23 neutralizing antibodies to the cell cultures where PBMCs were stimulated with CA and SC. As shown in **Figure 5D**, MAIT cell proliferation was significantly reduced in the presence of anti-IL-23 in cultures stimulated with CA or SC extracts.

## Discussion

Recent lines of evidence revealed that some bacterial components may be involved in autoimmune responses (40, 41). However, currently, the role of the gut mycobiota in MS has been poorly investigated, mostly exploring the association between disease and *Candida* spp. (42). The observed differences in abundance and richness of cultivable gut mycobiota in our cohort of MS compared to HD allowed us to hypothesize that, in pwMS, fungi may indeed play functional roles in regulating immune responses.

Considering that, by culture-based analysis, the most frequently isolated fungal genera were *Candida* sp. sp. and *Saccharomyces* sp. sp., we investigated the abundances of the two main genera in metataxonomic data in the two cohorts.

Comparing the results obtained by the culture-based approach with metagenomic data on the characterization of fungal populations of the human gut, we detected some discrepancies deriving by methodological differences of the two procedures, as previously observed (29). This suggests that some fungal taxa identified by the metagenomic approach are lost with a culture-dependent approach and that a low percentage of the fungal population is cultivable with standard methods. These differences are due to culture conditions or because environmental fungi cannot survive through the GI tract, but their DNA is still detectable through molecular studies. Therefore, metagenomics analysis detected a fungal community structure that cultivation did not identify, but not at a deeper level than genus. On the other hand, culture-based analysis allowed us to obtain species-level information and to discern fungal phenotypes that would be otherwise lost by metagenomics. The fact that the fungal abundance measurements based on cultivation and targeted metagenomics are not identical probably reflects amplification biases that are known to be a major source of poor estimates of community abundance. These biases mainly derive from inconsistent amplification of the barcoding regions of different species, caused by copy number variations and different primer binding specificities (43, 44). The combination of the culturable yeast species component and the metagenomic approach based on ITS sequencing benefit one from the other and are complementary approaches, according to evidence produced from our group and others and thus can be considered the best way to quantify yeasts from the human gut (45, 46).

Strikingly, both the analyses in pwMS exhibit a much higher rate of food-associated strains shown to survive passage through the human gut, in particular *S. cerevisiae*, that here represents 6.5% of the total isolates in MS, compared to a known frequency of less than 2% in HD (29), and *S. delbrueckii*, another food-borne yeast (47–49). Other recent studies (13, 50) have shown alterations in the gut mycobiota of NEDA pwMS, but it is not yet clear if further alterations exist during the active phase of the disease.

The overall results thus show clonal expansion and increase in abundance of strains from common fungal species in the pwMS, yet it is noteworthy that some normal commensal species are less frequent and less abundant in pwMS than in normal individuals, thus suggesting that the immune response of pwMS to fungi is different from that of HD.

Other experimental data suggest a connection between GI immune responses and CNS autoimmunity: (i) alterations of gut microbiota composition change the outcome of experimental autoimmune encephalomyelitis (EAE) (51); (ii) in the small intestine, pro-inflammatory cells acquire a regulatory phenotype and suppress EAE (52); (iii) autoreactive cells of the CNS are activated in the gut-associated lymphatic tissue (53); and (iv) T cells, able to regulate EAE, expand in the gut and are found in MS lesions (54).

Furthermore, antibodies against GI antigens may indicate altered microbiota and immune responses in the gut. A study showed that anti-gliadin and anti-*S. cerevisiae* antibody (ASCA) were more frequent in patients affected by AQP4-seropositive neuromyelitis optica compared to controls (55).

Thus, the intellectual framework we have developed suggests that increased levels of fungal antigens, probably cell wall-related, present in different fungal species, converge to the activation of the mounting and the memory of an uncontrolled immune response potentially ending up in autoimmunity in pwMS. This would explain why, rather than an enrichment of the same species, similar and taxonomically related species often present as food-borne commensals are enriched in pwMS with respect to controls. The fungal enrichment is rather causal than consequential, since the enhanced Th1 and Th17 responses in pwMS should lead to fungal clearance, rather than to fungal invasion. It is probable that a leaky gut, or a combination of genetic, immune history, and fungal control factors, contributes to the onset of disease, and its observed association with enrichment in otherwise GRAS fungal species (Generally Recognized As Safe), like Ascomycetes of the *Saccharomyces* genus, as well as the usual culprit, the commensal but opportunistic pathogen *C. albicans*, previously associated to pathogenicity in several diseases (30), which was previously shown to express ASCA epitopes on mannoproteins similar to those of *S. cerevisiae* (56, 57).

In order to draw causality from these correlations, we explored the possibility that *S. cerevisiae* and *C. albicans* gut isolates could act in CNS autoimmunity. Thus, we selected two isolates of *S. cerevisiae* (namely, *SC2* and *SC12*) and two isolates of *C. albicans* (namely, *SM106* and *SM45*) with different phenotypic characteristics (**Table 2**), in order to perform immunological studies.

As an initial screen to test for immune reactivity to these strains, we measured induction of CD69 expression, a T-cell activation marker, in freshly isolated PBMCs from HD and pwMS cultured with SC and CA extracts. Flow cytometry data indicated that, indeed, T lymphocytes upregulate CD69 expression following challenge with these fungi, and that in pwMS, MAIT cells in particular become activated and proliferate. A previous study has shown reactivity of MAIT cells to CA and SC exposure (17), but here we measured responses to strains derived directly from the samples from pwMS. MAIT cells usually reside in the intestinal mucosa, where they cooperate with other immune cells in the control of the local microbiota. We and others have previously shown that, in MS, this cell population is altered in frequency in both the relapsing remitting and progressive form of the disease (31, 58, 59). Also, it was shown that MAIT cells are decreased in frequency in pwMS, and an inverse correlation was found between Tc17 MAIT cells in the peripheral blood and CNS demyelination (60), supporting the hypothesis that during active disease, MAIT cells are recruited in the CNS, and disappear from the circulation.

The finding that the fungal strains isolated from pwMS preferentially and significantly activate MAIT cells was then further investigated, and functional immunological studies were performed. What may determine this proliferation? We find that IL-23 released by monocytes following encounter with fungi selectively stimulates MAIT cells, independent of TCR engagement. Indeed, similar to natural killer (NK) cells, MAIT cells have been shown to be activated by cytokines such as IL-12 and IL-18 produced by innate immune cells during bacterial infections (39, 61), even in the absence of TCR ligation. Interestingly, the gene for the IL-23 receptor (IL-23R) has been shown to be associated with MS, and high levels of serum IL-23 have been detected in pwMS (62). Moreover, we find that MAIT cells from pwMS express higher levels of the IL-23R compared to healthy controls, making them more responsive to the proliferative stimulus delivered by IL-23.

**Figure 6:**
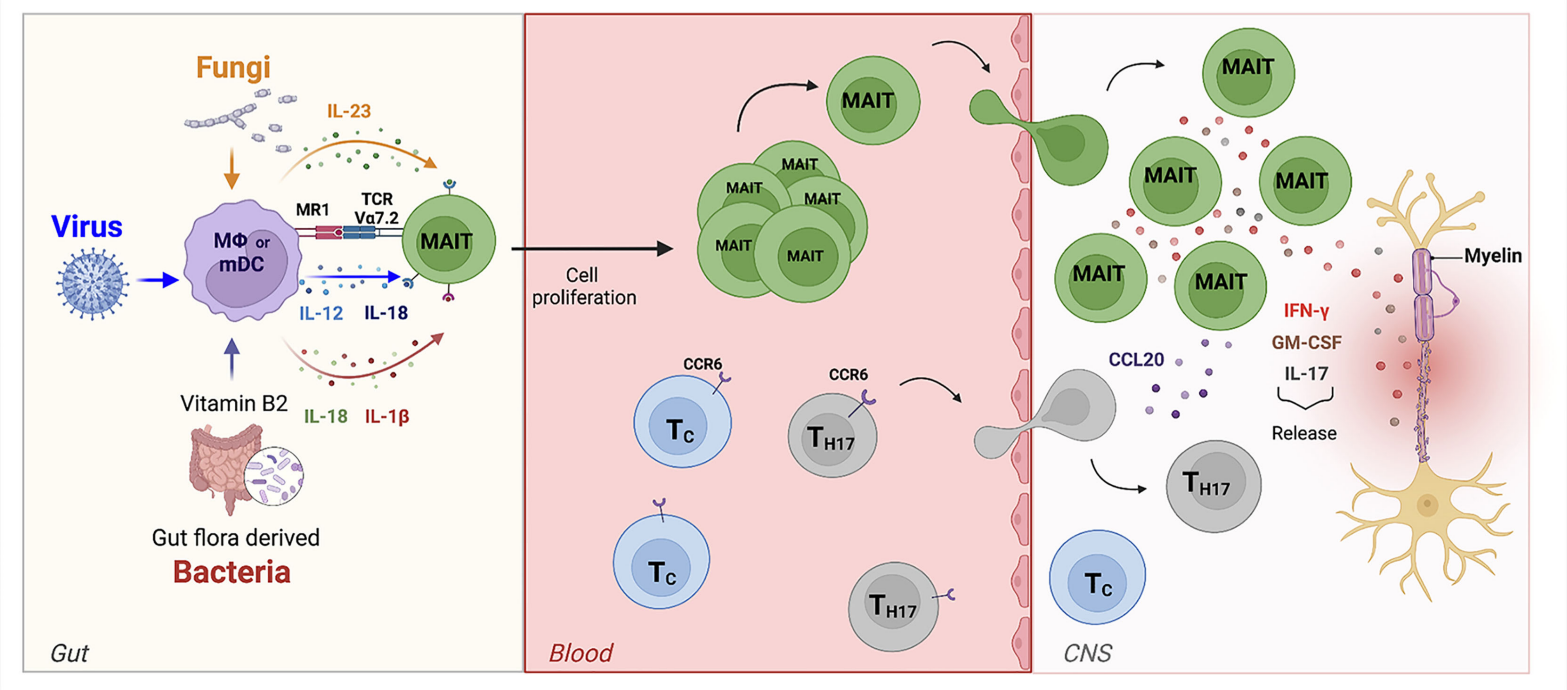
The role of MAIT cells in MS. In the gut, bacteria, viruses, and fungi activate MAIT cells in TCR-dependent or independent manner. MAIT cells then migrate towards the CNS. Here, the release of pro-inflammatory cytokines such as IL-17, GM-CSF, and IFN-g by MAIT cells contribute to axonal and neuronal damage. They are also able to release the chemokine CCL20, which amplifies CNS inflammation by recruiting of other CCR6-expressing-T cells from the periphery across the BBB into the CNS.

The final scenario provided by our data (**Figure 6**) proposes a central role for MAIT cells in MS pathogenesis where these unconventional T cells can be activated by different dysbiotic microorganisms in both a TCR-dependent and a TCR-independent manner. Viruses may lead to MAIT cell activation through innate immune-mediated release of IL-12 and IL-18, while activation induced by bacteria and fungi may involve an MR1-restricted response to riboflavin metabolites or a TCR-independent activation mediated by the release of IL-23 by innate immune cells. Interestingly, MAIT cells producing IL-17 together with IL-23 (an IL-17/Il-23 axis) have been proposed to participate as a major mechanism of disease in other autoimmune diseases (60, 63). These cells become pathogenic upon IL-23 activation (released by dendritic cells and monocytes) and, once they migrate to the CNS, they initially produce IL-17 and then GM-CSF and IFN-γ thus participating to the autoimmune reaction (64). Similar features (high expression of CD161, expression of CCR6 and production of IL-17) were found in γδ T cells enriched in the cerebrospinal fluid (CSF) of pwMS (64); γδ T cells producing IL-17 have also been found to be pathogenic in another autoimmune-mediated disease, psoriasis, again in response to IL-23 (65). Lastly, a recent hypothesis postulates that external perturbation to early-life microbiota can induce long-term microbial dysbiosis and promote proinflammatory unconventional T cells, such as MAIT and γδ T cells, and contribute to autoimmunity (21).

Thus, this study, combined with data from existing literature, confirms role for MAIT cells as central sensors at mucosal barriers, able to respond to different types of pathogens through the production of cytokines and chemokines that instruct the ensuing immune response and which may participate in creating the inflammatory milieu at the basis of CNS autoimmunity.

## Materials and methods

### Study participants and samples’ handling and collection

Blood samples were collected from a total of 49 pwMS (24 men, 25 women; average age, 42.7 years; range, 30–55 years; EDSS 0–3.5) with a clinical diagnosis of the relapsing–remitting form of multiple sclerosis (RR-MS) established according to the McDonald’s criteria (McDonald, 2017) and from 30 HD (HD; 15 women, 15 men; average age, 40.3 years, range, 27–54 years), into 20-ml sodium heparin Vacutainer tubes (BD Biosciences, San Jose, CA) at the Multiple Sclerosis Centre of the IRCCS Fondazione Santa Lucia, Rome, Italy, at the Neurology Department of the San Camillo Hospital, Rome, and at the Multiple Sclerosis Centre of the Hospital of Sant’ Andrea, Sapienza University, Rome, Italy, according to the guidelines and recommendations of the three institutions that approved the study. All patients were free of treatment for at least 6 months (some patients were naïve and some in the washout period between treatments). At the time of sampling, 21 pwMS fell in the EDA (evidence of disease activity) and 28 fell in the NEDA (no evidence of disease activity) category, following evaluation of clinical and neuroradiological parameters. Among this group of donors, 4 pairs of monozygotic twins discordant for MS were also included: 4 MS twins and 4 HD twins. All pwMS and HD provided informed consent in compliance with national legislation and the Code of Ethical Principles for Medical Research Involving Human Subjects of the World Medical Association (Declaration of Helsinki). Stool samples were collected from 27 NEDA pwMS and 21 age- and sex-matched HD and stored at −80°C until analysis. Metagenomic analyses were performed in 20 samples from NEDA pwMS and 18 samples from HD. All subjects selected for the study of mycobiota were under a Mediterranean-based diet, and no antibiotics, probiotics, or prebiotics have been taken in the 3 months prior to the sample collection. For the immunological studies, PBMCs from HD and RR-MS were isolated by Ficoll and immediately stained for flow cytometric analysis.

### Isolation and identification of cultivable fecal fungi

Stool samples were diluted in physiologic solution and plated on solid YPD medium (1% Yeast extract, 2% Bacto-peptone, 2% D-glucose, 2% agar) supplemented with 25 U/ml of penicillin and 25 μg/ml of streptomycin (Sigma-Aldrich) and incubated aerobically at 27°C for 3–5 days. All fungal isolates grown on the selective medium were further isolated to obtain single-cell pure colonies. From pure cultures of isolated colonies, genomic DNA was extracted and strains were identified by amplification and sequencing of the ribosomal Internal Transcribed Spacer (ITS1-5.8S-ITS2) region, as previously described by Sebastiani et al. (66) and Strati et al. (29). Fungal isolates were identified by using the BLAST algorithm in the NCBI database (minimum 97% sequence similarity and 95% coverage with a described species).

In order to evaluate different abundances of fungal species between pwMS and HS groups, abundance correction was performed taking into account the number of subjects in which a fungal species was isolated. Thus, we calculated the frequency of fungal isolates belonging to a certain species in the pwMS or HD cohort as the ratio between number of subjects with fungal isolates and the total number of subjects in a cohort. Then, the frequency of fungal isolates was multiplied by the number of isolates obtained from those subjects.

In this way, the corrected abundance positively varies in relation to the frequency of isolation, as frequently finding a certain species in a group can be considered as a confirmation of the count data, and negatively varies with frequency of isolation, penalizing count data in subjects with a single isolated fungal species.

### DNA extraction and PCR amplification of fungal ITS1 rDNA region

Total DNA extraction from fecal samples (250 mg) was performed using the Fast DNA TM SPIN Kit for Feces (MP-Biomedicals, USA) following the manufacturer’s instructions. For each sample, a primer set specific for fungal ITS1 rDNA region (18SF: 5′-GTAAAAGTCGTAACAAGGTTTC-3′ and 5.8S1R: 5′-GTTCAAAGAYTCGATGATTCAC-3′) were used (67). These primers were coupled with forward and reverse primer containing adaptors, key sequence, and barcode (Multiple IDentifier) sequences as described by the 454 Sequencing System Guidelines for Amplicon Experimental Design (Roche, Switzerland). The PCR reaction was performed as described by Strati et al. (29).

### Library construction and pyrosequencing

The PCR products obtained were analyzed by gel electrophoresis and cleaned using the AMPure XP beads kit (Beckman Coulter, USA) following the manufacturer’s instructions, quantified *via* quantitative PCR using the Library quantification kit—Roche 454 Titanium (KAPABiosystems, USA) and pooled in an equimolar manner in a final amplicon library. The 454 pyrosequencing was carried out on the GSFLX+ system using the XL+ chemistry following the manufacturer’s recommendations (Roche, Switzerland). Raw 454 data were demultiplexed using the Roche’s sff file software.

### Phenotypical characterization of fungal isolates

The subset of fecal fungal isolates reported in **Table 2** was tested for virulence-related traits, as reported in (29, 68).

#### Invasive growth

The ability of fungal isolates to penetrate solid-rich medium [YPD (yeast peptone dextrose), 1% yeast extract, 2% peptone, 2% glucose, and 2% agar) was tested to measure the potential ability of yeast cells to invade human tissues. Yeast cells (10^4^ and 10^2^ cells in 1 ml of sterile water) were spotted on solid YPD plates and incubated at 28°C and 37°C. After 5 days of growth, cultures were washed out and the remaining cells (invading the medium) were stained with Coomassie blue as previously described by Strati et al. (29) and Palková et al. (69).

#### Resistance to oxidative stress

Fungal resistance to oxidative stress was evaluated by measuring the inhibition halo resulting from treating fungal isolates (10^7^ cells in 1 ml of sterile water) spread onto YPD medium and adding in the plate center a sterile paper disk saturated with tert-Butyl-hydroperoxide (tBut-OOH 1 M). After 5 days of incubation at 28°C, the inhibition halo on YPD+ tBut-OOH 1 M was measured. The percentage of sensitivity to oxidative stress was calculated, measuring the inhibition halo diameter as described by Strati et al. (29).

#### Growth at supra-optimal temperatures

Fungal strains were grown at supra-optimal temperatures, to test a typical feature of clinically isolated and pathogen strains. Yeast cells (10^6^ cells in 1 ml of sterile water) were spotted on solid YPD medium and incubated at 4°C, 40°C, 42°C, and 44°C for 3 days.

#### Pseudohyphal and hyphal formation

Yeast cells (10^6^ cells/ml) were grown in liquid YPD and YNB media [0.67% Yeast Nitrogen Base w/o amino acids and (NH_4_)_2_SO_4_, 2% glucose], both at 28°C and 37°C. After 7 days of incubation, pseudo hyphae and hyphae formation were observed at microscopy.

#### Sporulation rate

A large amount of pre-aged fungal cells grown on YPD was transferred onto Spo IV (Sporulation IV medium: 2% potassium acetate, 0.25% yeast extract, and 0.1% glucose) and incubated at 4°C, 28°C, and 37°C. After 5 and 10 days, spore formation was assessed. The SK1 *S. cerevisiae* strain (ATCC:204722) was used as a positive control (100% sporulation efficiency). The sporulation percentage was calculated as the ratio of the number of asci observed on the total number of cells and asci.

### Metagenomic data analysis

After reads pre-processing (amplification primer and short reads removal) and denoising [performed using Acacia software (70)], the QIIME pipeline (v. 1.9, http://qiime.org//) was used for analysis (71). Chimeric reads were identified using the usearch61 method (72) and removed prior to OTUs picking, which was performed with the command *pick_open_reference_otus.py*, using an identity threshold of 97% with the uclust method (72). Reference database for fungal (ITS1) data was the UNITE ITS database (ver. 7) with the “dynamic” similarity threshold (73). Taxonomical assignment for fungal data was performed with the Blast method.

Data analysis was performed using R statistical software (Team, R. Core 2014). The phyloseq (74) and microbiome (75) packages were used for handling QIIME data in R and for Alpha and Beta diversity analysis (within-sample richness, Shannon’s index, and PCoA ordination). Prior to diversity analysis, data were normalized by Cumulative sum scaling (CSS). Species-level OTUs abundance changes between HD and MS subject groups were detected using the package DESeq2 (76) following indications in (77). Briefly, samples were not rarefied and counts were not CSS transformed; instead, normalization was achieved with the DESeq2’s variance-stabilizing transformation, and the abundance between MS and HD subjects’ group was then compared using Wald negative binomial test.

### Flow cytometry: Antibodies and reagents

To define lymphocyte activation and phenotype, polychromatic flow cytometry was used. PBMCs from pwMS and HD were stained with a cocktail of monoclonal antibodies conjugated to different fluorochromes: Vα7.2 FITC or BV421 (Clone 3C10, BioLegend), CD8 PercPCy5.5 (Clone SK1, Becton Dickinson), CD161 PE or APC-Alexa750 (Clone 191B8, Beckman Coulter), CD69 BV421 (Clone FN50, Becton Dickinson), CD3 PE-Cy7 (Clone UCTH1, Beckman Coulter), CD4 BV605 (Clone RPA-T4, Becton Dickinson), and IL-23R PE (Clone 218213, R&D Systems); to exclude dead cells, LIVE/DEAD Fixable Aqua Dead Cell Stain Kit (Invitrogen) was used. For intracellular staining, cells were first surface-stained with the antibodies indicated above, then fixed and stained intracellularly using the following antibodies: IFNγ APC (Clone B27, Becton Dickinson) and IL-17 Alexa 488 (Clone BL168, BioLegend).

Stained cells were acquired on a CytoFLEX flow cytometer (Beckman Coulter), equipped with five lasers and able to measure up to 23 parameters simultaneously on each cell. For each sample, approximately 300,000 lymphocytes were selected based on scatter parameters, and the analysis was conducted after the exclusion of dead cells and coincident events. The data were compensated and analyzed using FlowJo v10.6.1 (TreeStar, Ashland, OR). In some experiments, cell sorting with MoFlo Astrios (Beckman Coulter) was performed to obtain a pure population of MAIT cells. Purity of sorted cells was consistently >98%.

### Proliferation assays

PBMCs obtained from HD and pwMS were labeled with CFSE (5,6-carboxyfluorescein diacetate succinimidyl ester) 1 μg/ml (Molecular Probes), then seeded at 3 × 10^5^/well in RPMI 1640, and stimulated with different conditions: fungal extracts *C. albicans* (CA) from the fecal sample of pwMS (0.1 μl of 1 × 10*^8^* cells/ml), fungal extracts *S. cerevisiae* (SC) from the fecal sample of pwMS (0.1 μl of 1 × 10^8^ cells/ml), IL-23 (20 ng/ml, Miltenyi), or IL-23 neutralizing antibodies (10 μg/ml, R&D Systems). After 5 days, cells were collected and analyzed by flow cytometry.

### Cytokine detection in culture supernatants

Pure populations of MAIT cells (1 × 10^5^cells/well) were cultured in RPMI 1640 complemented with penicillin and streptomycin (100 μg/ml and 100 μg/ml, respectively), L-glutamine (2 mM), and 10% autologous human serum and stimulated with Dynabeads coated with mAb to CD3 plus mAb to CD28 (0.2 beads per cell) (Invitrogen). *In vitro* stimulation was performed at 37°C, 5% CO_2_, and after 72 h, cell culture supernatants were collected. Cytokine secretion was measured using the Luminex Human Magnetic Assay (16-Plex) (R&D Systems) following the manufacturer’s protocol. This assay allowed the simultaneous detection of the following cytokines: MCP-1/CCL2, MIP-1alpha/CCL3, MIP-1beta/CCL4, MIP-3/CCL20, GM-CSF, IL-15, IL-2R alpha, TNF-RI, TNFα, IL-10, IFN-γ, IL-17A, IL-6, IL-1beta, IL1 ra/IL-1F3, and IL-2. The analysis was performed with a Luminex Bio-plex 200 system (Life Technologies). In another set of experiments, monocytes were purified from PBMCs of HDs and pwMS with the Pan Monocyte Isolation Kit (Miltenyi), and then pulsed *in vitro* (1 × 10^5^cells/well) with the fungal strains CA and SC in culture medium at 37°C. After 70 h of stimulation, supernatants were collected. IL-23 production was measured using the IL-23 Human Uncoated ELISA Kit with Plates (ThermoFisher, Invitrogen). Assays were run according to the manufacturer’s protocols with a detection limit of standard curve range: 16 pg/ml to 2,000 pg/ml. Absorbance measurements were carried out by a Multiscan Ex reader (Thermo Scientific) at 450/640 nm.

### Statistical analysis

Statistical analyses for continuous variables were performed using the Student’s *t*-test after confirming normal distribution (GraphPad Prism application, v6.2). Statistical significance was inferred for *p*-values below 0.05.

### Immunohistochemical studies

This study was performed on human post-mortem brain tissues from 15 donors (22 tissue blocks) with secondary progressive MS and 2 donors who died without neurological diseases (3 tissue blocks), provided by the UK Multiple Sclerosis Tissue Bank at Imperial College London (https://www.imperial.ac.uk/medicine/multiple-sclerosis-and-parkinsons-tissue-bank). Based on the available clinical documentation, no immunotherapy is reported for the MS donors in the 6 months before death. Use of human tissue for research purposes was approved by the Ethics Committee of Istituto Superiore di Sanità (CE 12/356). MS cases analyzed were selected for the presence of a robust meningeal and white matter (WM) inflammation and/or areas of active demyelination in WM. Neuropathological assessment was performed as previously described (68, 78). Abdominal lymph nodes (provided by Dr. Egidio Stigliano, Policlinico A. Gemelli, Rome, Italy) were used as positive controls.

Air-dried 10-µm-thick cryosections cut from MS and control snap-frozen cerebral tissue blocks were analyzed by double and triple immunofluorescent stainings using the following specific antibodies: rabbit polyclonal anti-CD8 (Thermo Fisher Scientific, Waltham, MA, USA), mouse anti-CD161 IgG2a (clone 191B8, Beckton Coulter, Marseille, France), mouse anti-TCR Vα7.2 IgG1,k (clone 3C10, BioLegend, San Diego, CA), rabbit anti-IL-17 (AbCam, Cambridge, UK), goat anti-CCL20 (R&D Systems, Inc, Minneapolis, MN, USA), rat anti-GM-CSF IgG2a,k (clone BVD2-21C11, BioLegend), and rabbit anti-IFNγ (Abcam). Briefly, air-dried sections were post-fixed for 10 min in acetone at 4°C, rehydrated in PBS, incubated first with 10% normal sera for 1 hour at room temperature (RT), and then with the appropriate combination of primary Abs overnight at 4°C. Sections were then incubated with a mixture of the appropriate Alexa Fluor 488-, 568-, and TRITC-conjugated goat and donkey secondary antibodies (Invitrogen) in PBS + 5% NG or ND serum for 1 h at RT. For detection of GM-CSF, biotinylated donkey anti-rat Ig followed by streptavidin TRITC-conjugated was used. Finally, sections were treated with an auto-fluorescence quenching solution, 10 mM CuSO_4_ in 50 mM ammonium acetate (pH 5), for 5 min at RT. After washing in distilled water, sections were sealed in Prolong Diamond anti-fade mounting medium with 4’,6’-diamidino-2-phenylindole (DAPI) (Thermo Fisher Scientific) and analyzed with a digital epifluorescence microscope (Leica Microsystem, Wetzlar, Germany). Negative control stainings were performed using Ig isotype controls and/or pre-immune sera.

#### Cell count

Following microscopic examination, multiple labeled cells were counted in the brain inflammatory infiltrates of the whole section areas (median value, 2.6 cm^2^; range, 2.0 to 4.0 cm^2^) at 20× and 40× magnification. The percentages of double- and triple-positive cells in the total population of brain-infiltrating CD8 T cells or Vα7.2+ cells were calculated.

## Data availability statement

The raw data supporting the conclusions of this article will be made available by the authors, without undue reservation.

## Ethics statement

The studies involving human participants were reviewed and approved by IRCCS Santa Lucia Foundation Ethical Committee and San Camillo Hospital Ethical Committee and S. Andrea Hospital, Sapienza University Ethical Committee. The patients/participants provided their written informed consent to participate in this study.

## Author contributions

LB, GB, CDF, and DA coordinated and supervised the work. FG, GG, EP, SD’O, and MDB performed all cellular biology and flow cytometry experiments. SR, CG, MCB, VA, RM, GR, and MS selected, recruited healthy donors and MS patients and performed all clinical evaluations. LR, LP, FV, DC, CB, and CF performed all culturomic and metagenomic analysis. BR and BS performed all Immunohistochemical studies. GG, CV, GB, CDF, DC, FV, BS, GR, and LB performed data analysis and wrote the manuscript. MP performed data analysis. LB and GB provided funding. All authors contributed to the article and approved the submitted version.

## Funding

This work was partially supported by the Italian Ministry of Health, Italy (cod. RF-2011-02346771 and RF-2018-12366111) to LB and (cod. RF-2016-02363688) to GB and by FISM Fondazione Italiana Sclerosi Multipla (Progetto Speciale 2018/S/5) to LB. MP is supported by a research fellowship FISM - Fondazione Italiana Sclerosi Multipla – cod. 2020/BR/012 and financed or co-financed with the ‘5 per mille’ public funding.

## Conflict of interest

The authors declare that the research was conducted in the absence of any commercial or financial relationships that could be construed as a potential conflict of interest.

## Publisher’s note

All claims expressed in this article are solely those of the authors and do not necessarily represent those of their affiliated organizations, or those of the publisher, the editors and the reviewers. Any product that may be evaluated in this article, or claim that may be made by its manufacturer, is not guaranteed or endorsed by the publisher.
