# Supplementary material for: Proinflammatory mucosal-associated invariant CD8+ T cells react to gut flora yeasts and infiltrate multiple sclerosis brain
Source: Front Immunol. 2022 Jul 28;13:890298. doi: 10.3389/fimmu.2022.890298 (PMC9376942; doi:10.3389/fimmu.2022.890298)
Supplement: Supplementary file 1 [file DataSheet_1.pdf]

Tab. 1

Species	n. isolates				n. subjects		Abundance value correction	
	MS	HD	% on total MS isolates	% on total HD isolates	MS	HD	MS	HD
Candida albicans	388	30	24.1%	7.7%	9	7	124.71	14.00
Candida parapsilosis	196	19	12.2%	4.8%	4	4	28.00	5.07
Saccharomyces delbrueckii	185	2	11.5%	0.5%	2	2	13.21	0.27
Saccharomyces cerevisiae	99	1	6.2%	0.3%	3	1	10.61	0.07
Pichia membranifaciens	200	0	12.4%	0.0%	1	0	7.14	0.00
Exophiala dermatitidis	76	50	4.7%	12.8%	2	1	5.43	3.33
Candida zeylanoides	123	0	7.6%	0.0%	1	0	4.39	0.00
Aspergillus pseudoglaucus	15	55	0.9%	14.0%	7	1	3.75	3.67
Candida glabrata	84	1	5.2%	0.3%	1	1	3.00	0.07
Exophiala phaeomuriformis	73	0	4.5%	0.0%	1	0	2.61	0.00
Pichia manshurica	70	12	4.4%	3.1%	1	2	2.50	1.60
Rhodotorula mucilaginosa	8	15	0.5%	3.8%	6	3	1.71	3.00
Penicillium commune	11	0	0.7%	0.0%	4	0	1.57	0.00
Aspergillus ruber	19	0	1.2%	0.0%	2	0	1.36	0.00
Galactomyces candidum	16	199	1.0%	50.8%	1	1	0.57	13.27
Trichosporon sp	12	0	0.7%	0.0%	1	0	0.43	0.00
Aspergillus terreus	2	1	0.1%	0.3%	2	1	0.14	0.07
Corynascus sepedonium	4	0	0.2%	0.0%	1	0	0.14	0.00
Pichia fermentans	3	0	0.2%	0.0%	1	0	0.11	0.00
Pyrenochaeta unguis hominis	3	0	0.2%	0.0%	1	0	0.11	0.00
Pichia kudriavzevii	2	0	0.1%	0.0%	1	0	0.07	0.00
Candida orthopsilosis	1	0	0.1%	0.0%	1	0	0.04	0.00
Chaetomium brasiliense	1	0	0.1%	0.0%	1	0	0.04	0.00
Chaetomium globosum	1	0	0.1%	0.0%	1	0	0.04	0.00
Cryptococcus albidus	1	0	0.1%	0.0%	1	0	0.04	0.00
Cryptococcus diffluens	1	0	0.1%	0.0%	1	0	0.04	0.00
Dipodascaceae family isolate	1	0	0.1%	0.0%	1	0	0.04	0.00

Geotrichum candidum	1	0	0.1%	0.0%	1	0	0.04	0.00
Malassezia globosa	1	0	0.1%	0.0%	1	0	0.04	0.00
Penicillium carneum	1	0	0.1%	0.0%	1	0	0.04	0.00
Penicillium chrysogenum	1	0	0.1%	0.0%	1	0	0.04	0.00
Penicillium echinulatum	1	0	0.1%	0.0%	1	0	0.04	0.00
Penicillium expansum	1	0	0.1%	0.0%	1	0	0.04	0.00
Penicillium infrapurpureum	1	0	0.1%	0.0%	1	0	0.04	0.00
Phoma sp	1	0	0.1%	0.0%	1	0	0.04	0.00
Talaromyces stollii	1	0	0.1%	0.0%	1	0	0.04	0.00
Thelebolus ellipsoideus	1	0	0.1%	0.0%	1	0	0.04	0.00
Thielavia sp	1	0	0.1%	0.0%	1	0	0.04	0.00
Trichosporon ovoides	1	0	0.1%	0.0%	1	0	0.04	0.00
Wickerhamomyces anomalus	1	0	0.1%	0.0%	1	0	0.04	0.00
Candida lusitaniae	0	1	0.0%	0.3%	0	1	0.00	0.07
Debaryomyces hansenii	0	1	0.0%	0.3%	0	1	0.00	0.07
Pichia kluyveri	0	1	0.0%	0.3%	0	1	0.00	0.07
Rhizopus oryzae	0	1	0.0%	0.3%	0	1	0.00	0.07
Rhodosporidium kratochvilovae	0	2	0.0%	0.5%	0	2	0.00	0.27
Trichosporon guehoae	0	1	0.0%	0.3%	0	2	0.00	0.13
**Total n. isolates**	**1608**	**392**						

Tab. 2

ID strain	Identification by ITS1-4 Sanger sequencing	Growth at supra optimal temperatures		Hyphal and pseudo-hyphal formation				Sporulation	Invasiveness	Oxidative stress (resistance)
				YNB		YPD				
		40°C	42°C	27°C	37°C	27°C	37°C			
#17 YMS24-1	*C. parapsilosis*	++	++	-	-	-	-	-	+++	+++
**#106 YMS102-11**	***C.albicans***	++	++	-	+	-	+	-	+++	+++
#128 YMS12-3	*C.albicans*	++	++	-	-	-	-	-	+++	++
#18 YMS24-2	*C.albicans*	++	++	-	-	-	-	-	-	-
#25 YMS25-7	*C.albicans*	++	++	+	-	+		-	-	+++
#32 YMS25-14	*C.albicans*	++	++	-	+	-	+	-	+++	+
#38 YMS25-20	*C.albicans*	++	++	-	+	-	+	-	+++	+++
**#45 YMS25-27**	***C.albicans***	++	++	-	-	-	-	-	-	+
#51 YMS27-5	*C.albicans*	++	++	-	-	-	-	-	-	+++
#6 YMS19-6	*C.albicans*	++	++	-	-	-	-	++	-	++
#63 YMS28-7	*Cryptococcus albidus*	-	-	-	-	-	-	-	-	+
#120 YSM-8-2	*Debaryomyces hansenii*	++	++	-	-	-	-	-	-	+++
#119 YSM-8-1	*Pichia Kluvery*	++	++	-	-	-	-	-	-	+++
**#2 YMS19-2**	***S.cerevisiae***	++	++	-	-	-	-	++	-	++
#3 YMS19-3	*S.cerevisiae*	++	++	-	+	-	+	++	-	+
**#12 YMS20-2**	***S.cerevisiae***	+	-	-	-	-	-	-	++	+
#13 YMS20-3	*S.cerevisiae*	++	++	-	-	-	-	-	-	-
#14 YMS20-4	*S.cerevisiae*	++	++	-	-	-	-	++	+	+++
#136 YMS18-1	*S.cerevisiae*	++	++	-	-	-	-	++	+	-
#113 YMS7-1	*T.delbrueckii*	+	-	-	-	-	-	-	+	+ +
#114 YMS7-2	*T.delbrueckii*	++	++	-	-	-	-	-	-	++
#117 YMS7-5	*T.delbrueckii*	++	++	+	-	+ (pseudo hyphae)	-	++	-	+++
#118 YMS7-6	*T.delbrueckii*	++	++	-	-	-	-	-	-	+++
#115 YMS7-3	*T.ovoides*	++	++	+	+	+ (pseudo hyphae)	+ (pseudo hyphae)	-	+++	+++
#111 YMS1-1	*Trichosporon sp*	++	++	-	+	+ (pseudo hyphae)	+	-	+++	++
