# Supplementary material for: Proinflammatory mucosal-associated invariant CD8+ T cells react to gut flora yeasts and infiltrate multiple sclerosis brain
Source: Front Immunol. 2022 Jul 28;13:890298. doi: 10.3389/fimmu.2022.890298 (PMC9376942; doi:10.3389/fimmu.2022.890298)
Supplement: Supplementary file 2 [file Presentation_1.pptx]

## Slide 1
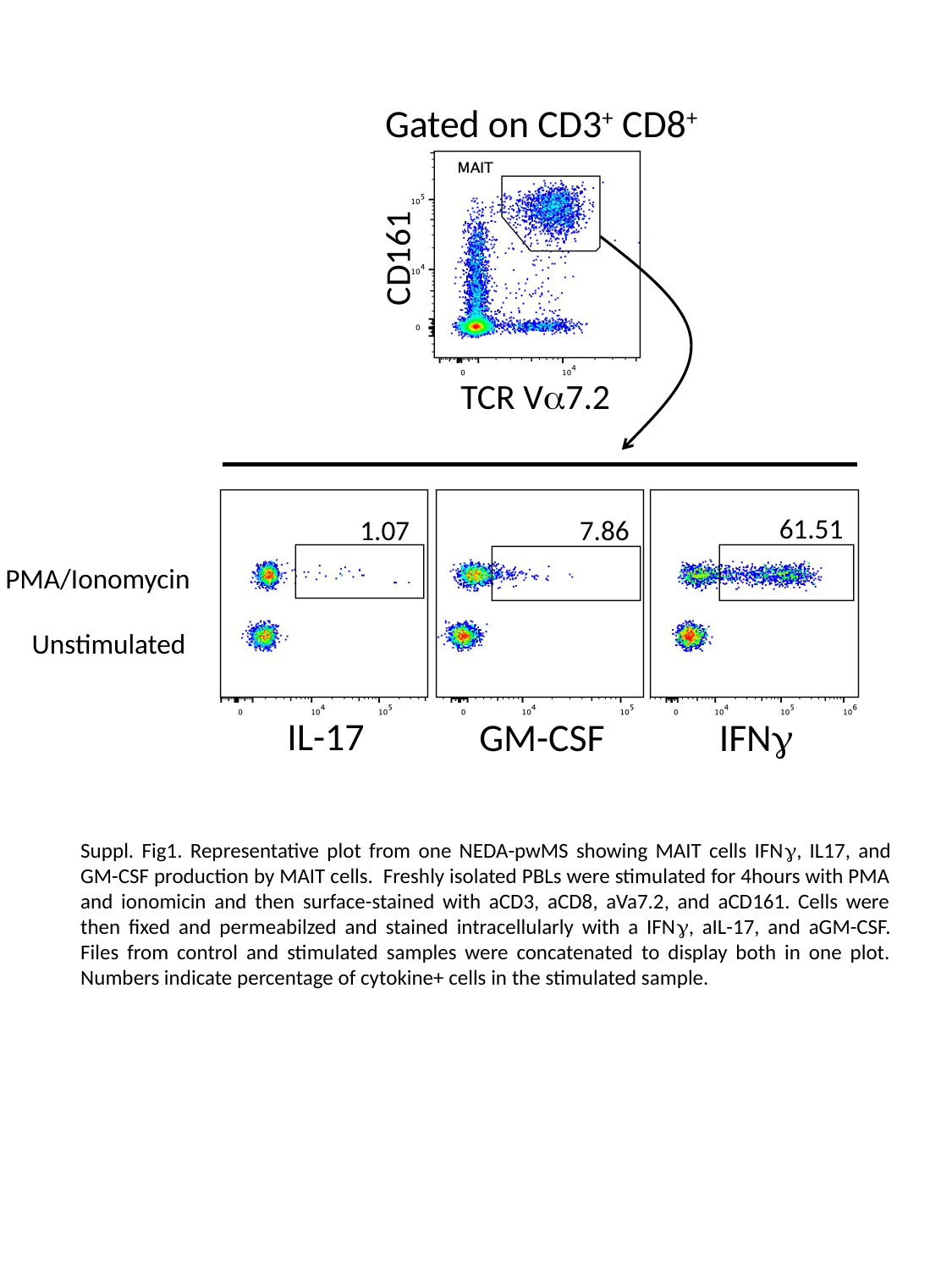

Gated on CD3+ CD8+
CD161
TCR Va7.2
IL-17
IFNg
GM-CSF
61.51
1.07
7.86
PMA/Ionomycin
Unstimulated
Suppl. Fig1. Representative plot from one NEDA-pwMS showing MAIT cells IFNg, IL17, and GM-CSF production by MAIT cells. Freshly isolated PBLs were stimulated for 4hours with PMA and ionomicin and then surface-stained with aCD3, aCD8, aVa7.2, and aCD161. Cells were then fixed and permeabilzed and stained intracellularly with a IFNg, aIL-17, and aGM-CSF. Files from control and stimulated samples were concatenated to display both in one plot. Numbers indicate percentage of cytokine+ cells in the stimulated sample.

## Slide 2
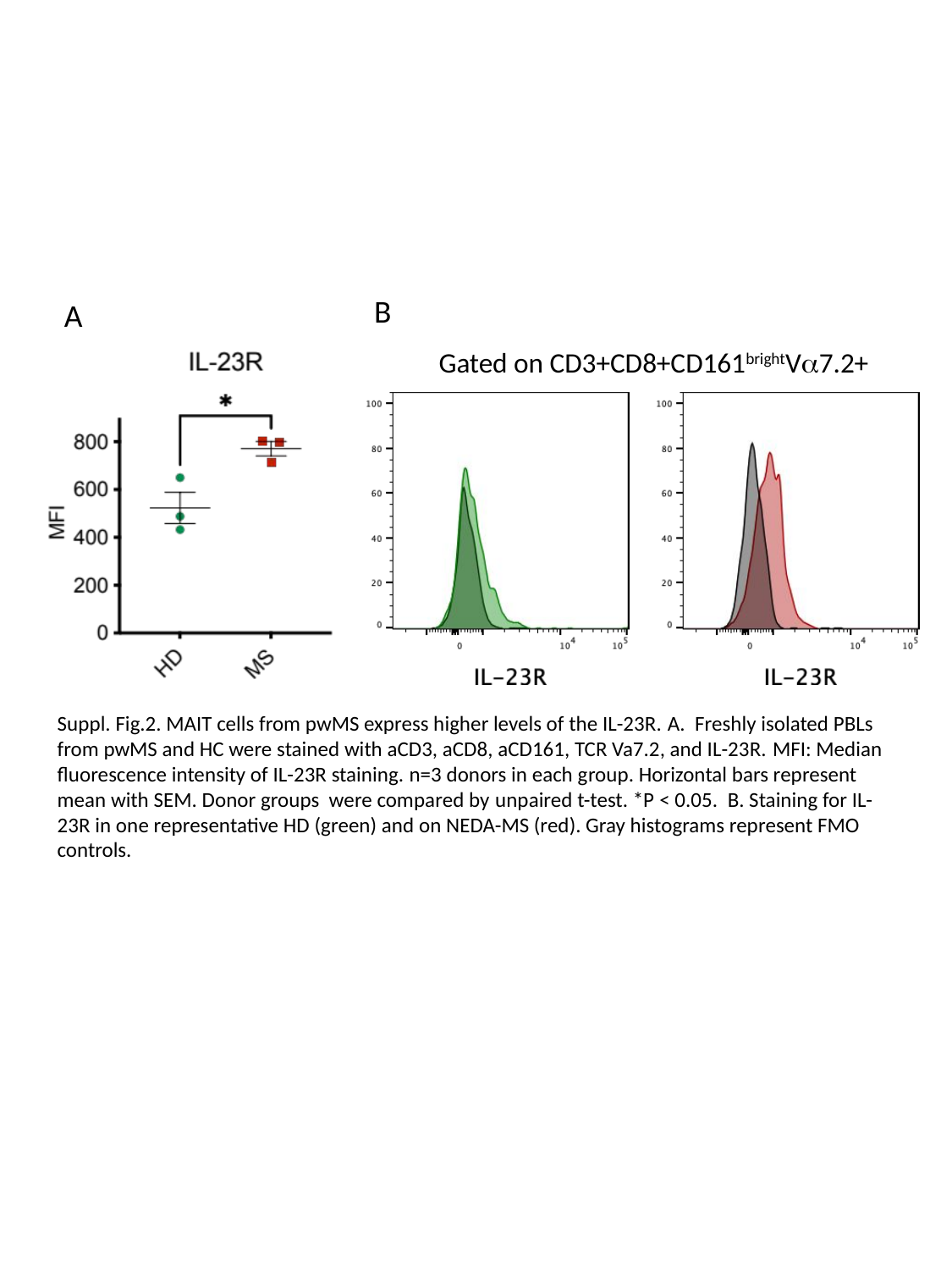

Gated on CD3+CD8+CD161brightVa7.2+
B
A
Gated on CD3+CD8+CD161brightVa7.2+
Suppl. Fig.2. MAIT cells from pwMS express higher levels of the IL-23R. A. Freshly isolated PBLs from pwMS and HC were stained with aCD3, aCD8, aCD161, TCR Va7.2, and IL-23R. MFI: Median fluorescence intensity of IL-23R staining. n=3 donors in each group. Horizontal bars represent mean with SEM. Donor groups were compared by unpaired t-test. *P < 0.05. B. Staining for IL-23R in one representative HD (green) and on NEDA-MS (red). Gray histograms represent FMO controls.
